# Japan nosocomial infections surveillance (JANIS): a model of sustainable national antimicrobial resistance surveillance based on hospital diagnostic microbiology laboratories

**DOI:** 10.1186/s12913-018-3604-x

**Published:** 2018-10-20

**Authors:** Atsuko Tsutsui, Satowa Suzuki

**Affiliations:** Antimicrobial Resistance Research Center, National Institute of Infectious Diseases, 4-2-1 Aoba-cho, Higashi-Murayama, Tokyo, 189-0002 Japan

**Keywords:** Antimicrobial resistance, Surveillance, Diagnostic microbiology laboratories, Comprehensive data collection

## Abstract

**Background:**

Antimicrobial resistance (AMR) is now recognized as a major threat to public health, and surveillance of AMR is essential for successful containment. In 2000, Japan Nosocomial Infections Surveillance (JANIS) Clinical Laboratory (CL) division has been launched as a voluntary AMR surveillance funded by the Ministry of Health, Labour and Welfare and managed by the National Institute of Infectious Diseases. In this study, we aimed to propose a model of sustainable national AMR surveillance which provides not only national AMR surveillance reports but also benchmarking reports to each hospital to facilitate infection control practices.

**Methods:**

JANIS CL division collects comprehensive specimen-based data complies with JANIS data format from participating hospitals each month. It had targeted only blood and cerebrospinal fluid samples but was expanded to all types of specimens in 2007 at revision of JANIS. The JANIS system interprets the antimicrobial susceptibility according to the same criteria and conducts removal of duplicates to allow accurate comparison between hospitals. Monthly feedback reports are created automatically within 48 h, while quarterly and annual reports are generated after data validation.

**Results:**

At the beginning, 468 hospitals were enrolled in the JANIS CL division, but the number of hospitals that submitted data decreased to 210 (45%) in 2006. After surveillance revision in 2007, annual recruitment of hospitals was initiated and as of 2015, 1475 hospitals participated, and 1461 (99%) of them submitted data throughout the year. Nationwide surveillance data collected over the past decade revealed that the prevalence of methicillin-resistant *Staphylococcus aureus* has decreased since 2008, and that its prevalence is higher in the western part of Japan, where the number of hospitals per capita is higher than in the eastern part.

**Conclusions:**

JANIS CL division serves a model of sustainable national AMR surveillance system. Comprehensive data for all specimens promotes understanding of the sampling frequency and prevalence of AMR. As a well-established system for providing rich information to guide action both locally and nationally, JANIS may also be utilized for sharing AMR data globally.

**Electronic supplementary material:**

The online version of this article (10.1186/s12913-018-3604-x) contains supplementary material, which is available to authorized users.

## Background

Antimicrobial resistance (AMR) is now recognized as a major threat to public health that limits patients’ treatment options for bacterial infections. In 2015, the 68th World Health Assembly adopted the Global Action Plan on Antimicrobial Resistance [1]. One of the five strategic objectives of the Global Action Plan is to strengthen knowledge through surveillance and research [2].

In Japan, methicillin-resistant *Staphylococcus aureus* (MRSA) was one of the first pathogen that made health professionals realize that AMR is a real-world threat. MRSA was prevalent in healthcare settings in Japan in the 1980s, especially in surgical wards, where it causes post-surgery nosocomial infections [3]. Consecutively, multidrug-resistant *Pseudomonas aeruginosa* also emerged and spread in hospitals across Japan. This led the Ministry of Health, Labour and Welfare (MHLW) to establish a surveillance system for nosocomial infections and AMR, the Japan Nosocomial Infections Surveillance (JANIS).

Common types of infectious disease surveillance are usually patient-based, with case definitions consisting of clinical diagnoses based on the symptoms and signs of the patient, often accompanied by diagnostic test results [4]. By contrast, AMR surveillance must focus on the isolated pathogen rather than the patient. Microbiological test results of bacterial identification and antimicrobial susceptibility testing (AST) are the primary components of AMR surveillance [5]. Therefore, many existing AMR surveillance systems rely on data generated by microbiology laboratories [6–9].

The main AMR surveillance component of JANIS is its Clinical Laboratory (CL) division, which collects data from diagnostic microbiology laboratories of hospitals. However, the outstanding characteristic of the Japanese medical system is that it has one of the largest number of hospitals in the world [10]. In 2014, there were 8493 Japanese hospitals, by far exceeding 5627 hospitals in the United States [11]. The large number of hospitals results in dispersed healthcare resources, and more than 1000 hospitals run their own microbiology laboratories. Therefore, a practical surveillance system was required to continuously accumulate data from those laboratories to achieve national surveillance. In this study, we aimed to propose a model of sustainable national AMR surveillance which overcame those barriers and creates not only national reports but also benchmarking reports for each hospital to facilitate infection control practices.

## Methods

### Administration and surveillance sites of the JANIS CL division

JANIS is a voluntary surveillance funded by the MHLW and managed by the National Institute of Infectious Diseases. It consists of five divisions; in addition to the CL division for AMR surveillance, there are divisions responsible for surveillance of health care–associated infection. The JANIS steering committee, which makes recommendations for improving the surveillance, consists of experts in field of infectious diseases, infection prevention and control, and clinical microbiology. The committee was organized in 2007 when the surveillance was revised.

Surveillance sites of JANIS CL divisions was fixed to 468 hospitals who were enrolled to the project at the time of launching. These sites are hospitals with 200 or more beds that mainly provide acute care and have their own laboratories for diagnostic microbiology testing. After the revision of JANIS in 2007, annual recruitment of large hospitals with 200 or more beds was initiated, and only in 2014, that recruitment of small hospitals with less than 200 beds began. Today, hospitals without their own microbiology laboratories can also participate in JANIS if subscribing commercial laboratories are able to provide surveillance data.

### Target data and data collection system

JANIS collects comprehensive specimen-based data from diagnostic microbiology laboratories. The type of target specimen was initially limited to blood and cerebrospinal fluids (CSF), but was subsequently expanded to all types of specimens in 2007. Because JANIS calculates the prevalence of AMR by defining the number of patients with specific resistant bacteria as the numerator, and the number of specimen-submitting patients as the denominator, both culture-positive results and culture-negative results are required. The number of specimen-submitting patients provides information on how frequent the samples are obtained from patients for microbiological investigation in each hospital. It enables to assess the bias on prevalence of AMR due to the variability in the frequency of culture sampling policy in participating hospitals. Specimens from active surveillance of hospitalized patients are included, but samples from the environment must be excluded from the surveillance data.

**Fig. 1:**
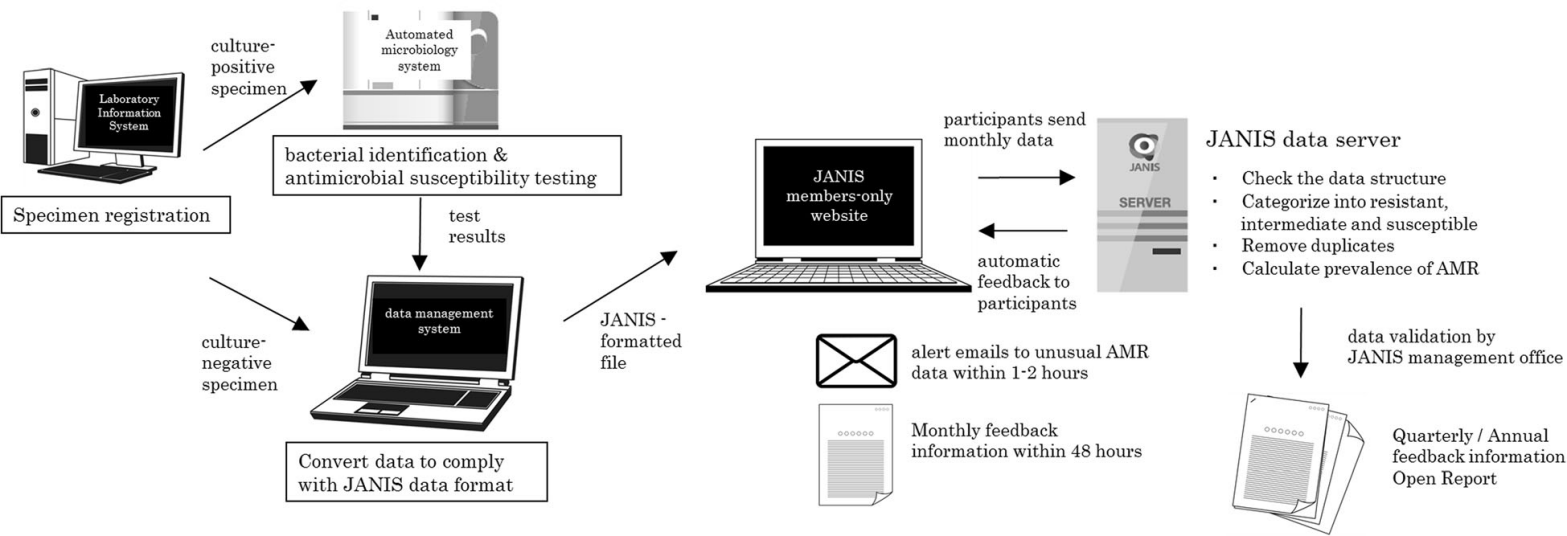
Data flow of JANIS Clinical Laboratory Division. For all clinical specimens tested, the JANIS system aggregates comprehensive bacterial data regarding patient, location, sample, organism, and antimicrobial susceptibility

Figure 1 shows the data flow of the JANIS CL division. Isolates from culture-positive specimens are usually tested by the automated microbiology system for bacterial identification and AST. Both culture-positive and -negative results must be integrated and converted into JANIS-formatted files.

The JANIS data format for CL division was developed as a unified standard for collecting electronic data from microbiology laboratories (Additional file 1). It defines information formats for patient demographics, sample type, isolated organism, and AST results, which are the key elements for AMR surveillance. Manufacturers of laboratory information systems have cooperated to provide an export function in their systems that complies with the JANIS data format.

### Data analysis for antimicrobial resistance

During the revision of the surveillance in 2007, JANIS activities became completely web-based and announcements from the surveillance are constantly updated on the website to provide necessary information for data submission. Each participant hospital submits surveillance data monthly by uploading the formatted file to the JANIS member–restricted website (Fig. 1). Submitted files from participating hospitals are automatically processed to check data structure, interpret antimicrobial susceptibility, remove duplicates, and calculate the prevalence of AMR. The files include data of outpatient samples, but data analysis is confined to inpatient samples.

To interpret AST results, minimal inhibitory concentrations (MICs) are categorized as resistant, intermediate, or susceptible (RIS) by the JANIS system. Interpretive criteria for antimicrobial susceptibility are mainly based on the Clinical and Laboratory Standards Institute (CLSI) guidelines, in combination with some of the reporting criteria for drug-resistant bacterial infections defined by the Infectious Diseases Control Law in Japan (Additional file 2) [12, 13].

Removal of duplicates is conducted to accurately calculate the prevalence of AMR. Identical resistant bacteria reported from the same patient within 30 days, regardless of specimen source, are excluded as duplicates. For antibiograms, removal of duplicates within 30 days is conducted based on AST results.

### Data validation

Participating hospitals can check for errors in data structure, warnings on ambiguous data, and microbiological alerts presented on the JANIS member–restricted website within a few hours after data submission (Fig. 1). Simultaneously, alert emails are automatically sent to the contact personnel of facilities reporting unusual combinations of bacterial species and antimicrobial susceptibility, defined as Unusual AMR (Additional file 3). Unusual AMR comprises two categories; Category A includes AMR that has never been officially reported in Japan such as vancomycin-resistant *Staphylococcus aureus*, whereas Category B includes AMR that have been reported but are still rare such as multidrug-resistant *Acinetobacter* spp. and vancomycin-resistant enterococci.

To validate quarterly and annual data, suspicious data such as data conversion errors (Additional file 4) and reports of Unusual AMR are checked by emails, postcards, and phone calls. If no response is obtained, all data from the hospital where the suspicious data originated are excluded from aggregation. Furthermore, a hospital will be expelled from JANIS if its data are excluded from annual reports for two consecutive years.

### Data feedback to the public and participating hospitals

Quarterly and annual Open Reports only include fully submitted hospital data. In addition to aggregated data from all hospitals, reports of data stratified by hospital bed counts (large [≥200 beds] and small [< 200 beds]) were generated starting in Annual Open Report 2014. Moreover, reports of data geographically stratified by 47 prefectures were generated starting from Annual Open Report 2015. The Open Reports are available on the JANIS website after data validation [14].

**Fig. 2:**
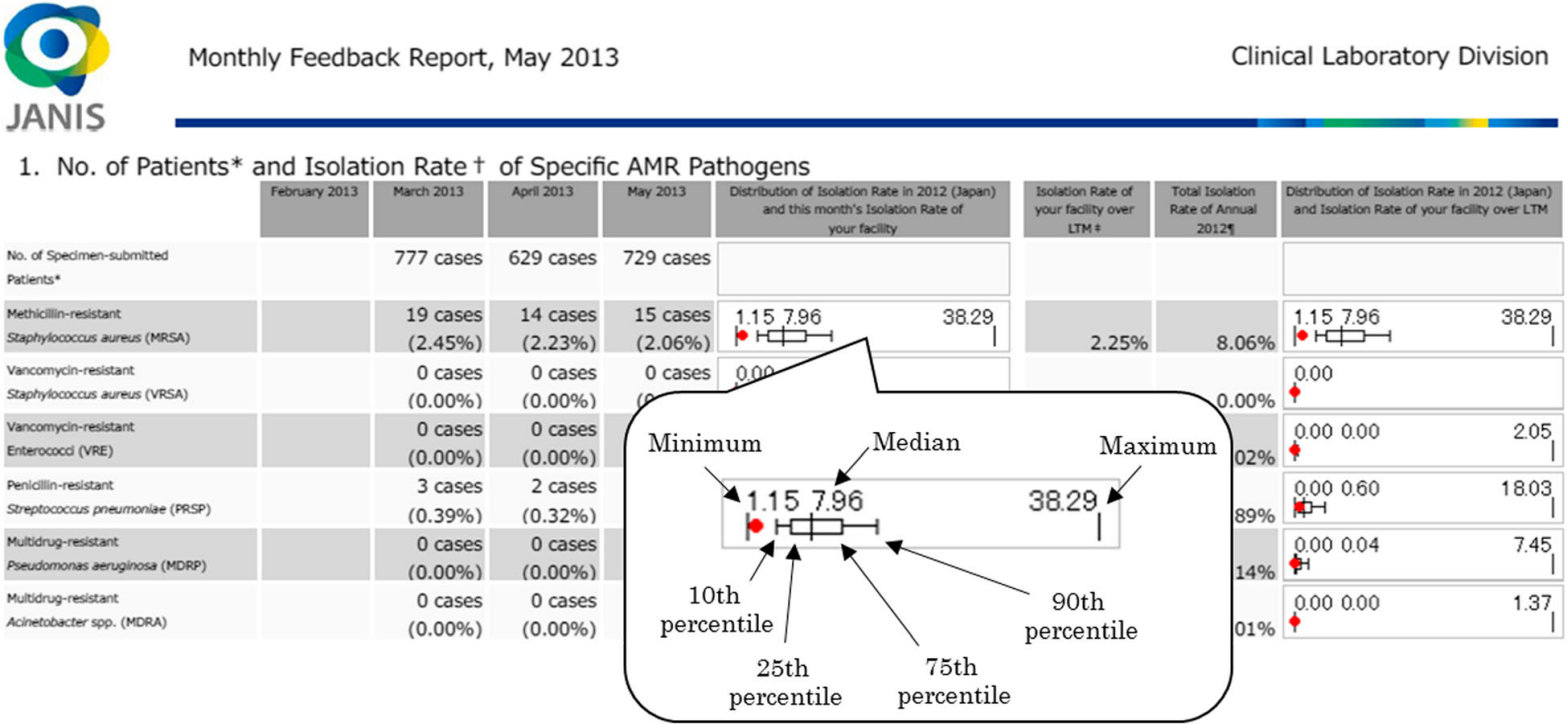
Box plot chart of JANIS. The box plot chart of JANIS represents the distribution of the prevalence of AMR of the facilities aggregated for the Open Report. Minima and maxima of all data, including outliers, are described at both ends of the graph, and whiskers represent 10th percentile and 90th percentiles. The (red) dot indicates the position of the corresponding facility in the entire distribution

Feedback to the participating hospitals, in the form of Feedback Reports, is produced for each hospital to guide and evaluate infection control practices. The main feature of the Feedback Reports is the original box plot chart (Fig. 2), which represents the distribution of the prevalence of AMR of the facilities aggregated for the Open Report. The red dot within the box plot indicates the position of the facility in the whole distribution of its hospital type (large or small). This red dot within the box plot enables benchmarking, allowing hospital to evaluate its prevalence of AMR and infection control performance against those of other facilities. Three types of Feedback Reports are available: monthly, quarterly, and annual. Monthly feedback information is automatically generated within 48 h of data submission in order to be useful to infection control committees in the facilities, whose meetings are typically held once a month. For annual Feedback Reports, in addition to the box plot charts on Open Reports stratified by hospital bed counts, data stratified by prefecture are also available. All of the Feedback Reports are available on the member-restrict JANIS web.

## Result

### Number of participating hospitals and representativeness

At the beginning of the surveillance in 2000, 468 hospitals were enrolled in the JANIS CL division, but the number of hospitals that submitted data was only 276 (59%) in 2001, decreasing to 210 (45%) in 2006. After the revision of the surveillance in 2007, due to annual recruitment and expansion of target hospitals, the number of JANIS member hospitals has been increasing constantly, reaching 928 by the beginning of 2014. In addition, because participation to JANIS CL division became one of the prerequisites for receiving additional reimbursement for infection control starting in April 2014, the number of member hospitals in 2015 increased dramatically, to 1.6 times the number in 2014. As of January 2017, there are 1840 JANIS member hospitals, which account for 21.7% of all hospitals throughout the country.

**Fig. 3:**
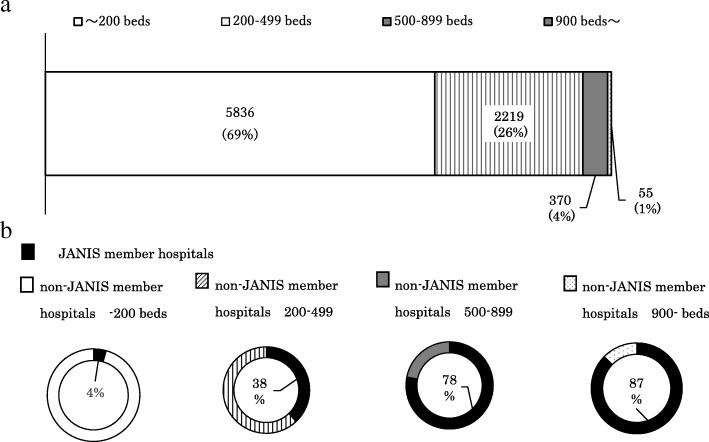
**a**) Number and proportions of hospitals, categorized by hospital bed count in Japan, according to Vital Statistics 2015. **b**) Proportion of JANIS member hospitals by bed counts for Annual Open Report 2015

Figure 3a shows the proportions of hospitals in Japan categorized by hospital bed count, and Fig. 3b shows the proportion of JANIS member hospitals in each category. In Japan, about 70% of hospitals have < 200 beds, and such facilities were not targeted by the JANIS CL division in 2000, when the surveillance was launched. Therefore, in 2015, about 80% of hospitals with ≥500 beds are covered by JANIS, whereas only 4% of small hospitals (< 200 beds) are covered. Thus JANIS CL division data may over-represent AMR epidemiology of large hospitals in Japan.

**Fig. 4:**
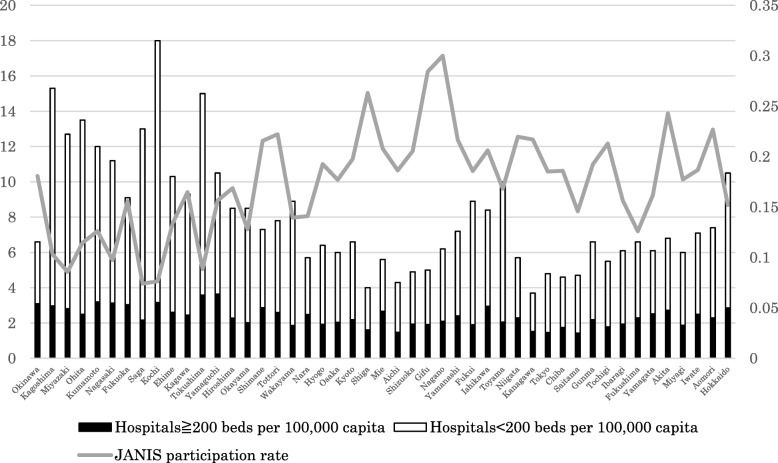
Proportion of JANIS member hospitals by prefectures for annual Open Report 2015. National data of facilities are according to Vital Statistics 2015

There are also geographical trends in JANIS participation. Figure 4 shows the proportion of JANIS participation and the number of hospitals per 100,000 capita by prefecture in 2015, ordered from left (southwestern part of Japan) to right (northeastern part). The proportion of JANIS participation among all hospitals varies by prefecture from 9.2 to 37.8% (median, 22.2%). Prefectures with low participation tend to have a larger number of hospitals per 100,000 capita, mainly consisting of small hospitals, and are especially concentrated in the western part of Japan.

### Data collection and analysis by the JANIS CL division

The number of specimens from which data was collected and analyzed for the Annual Open Report 2015 is shown in Table 1. In 2015, data were aggregated from more than seven million samples from 1435 JANIS member hospitals. On average, the number of specimens from large hospitals was 1361.6 per 100 beds, 2-fold more than from small hospitals (696.8 samples per 100 beds). The most frequent type of sample from large hospitals was blood (32.0%), followed by respiratory samples (28.4%) and others (18.7%). On the other hand, for small hospitals, respiratory samples were most frequent (36.6%) followed by blood (26.5%) and urine (15.8%). The proportion of culture-positive specimens from small hospitals was higher than that from large hospitals.

**Table 1:** Number of specimens in Annual Open Report 2015 by hospital bed counts

Specimen source	Hospitals ≥200 beds (*N* = 1177)		Hospitals < 200 beds (*N* = 258)	
	Number of specimens (%)	Proportion of culture-positive specimens	Number of specimens (%)	Proportion of culture-positive specimens
Respiratory	1,992,754 (28.4)	62.2%	99,273 (36.6)	70.9%
Urine	859,628 (12.3)	52.3%	42,697 (15.8)	67.6%
Feces	529,360 (7.5)	48.4%	18,950 (7.0)	49.3%
Blood	2,240,064 (31.9)	12.8%	71,851 (26.5)	15.8%
Cerebrospinal fluid	82,111 (1.2)	4.7%	1493 (0.6)	5.8%
Others	1,311,329 (18.7)	46.3%	36,617 (13.5)	48.7%
Total	7,015,246 (100.0)	40.5%	270,881 (100.0)	50.9%

### Data validation

In 2015, among 1475 participating facilities, 1461 (99.1%) submitted data thorough out the year. Approximately 400 alert emails were automatically sent to participants who reported Unusual AMR in 2015.

Among 152 hospitals undergoing validation, data from 26 facilities were excluded from aggregation; 12 hospitals were using only the disk diffusion method for AST, and 14 hospitals either did not correct suspicious data or did not respond to inquiries from the JANIS management office. Consequently, data from 97.3% (1435/1475) of the participating hospitals was aggregated for annual Open Report 2015.

### Prevalence of AMR in open report

**Fig. 5:**
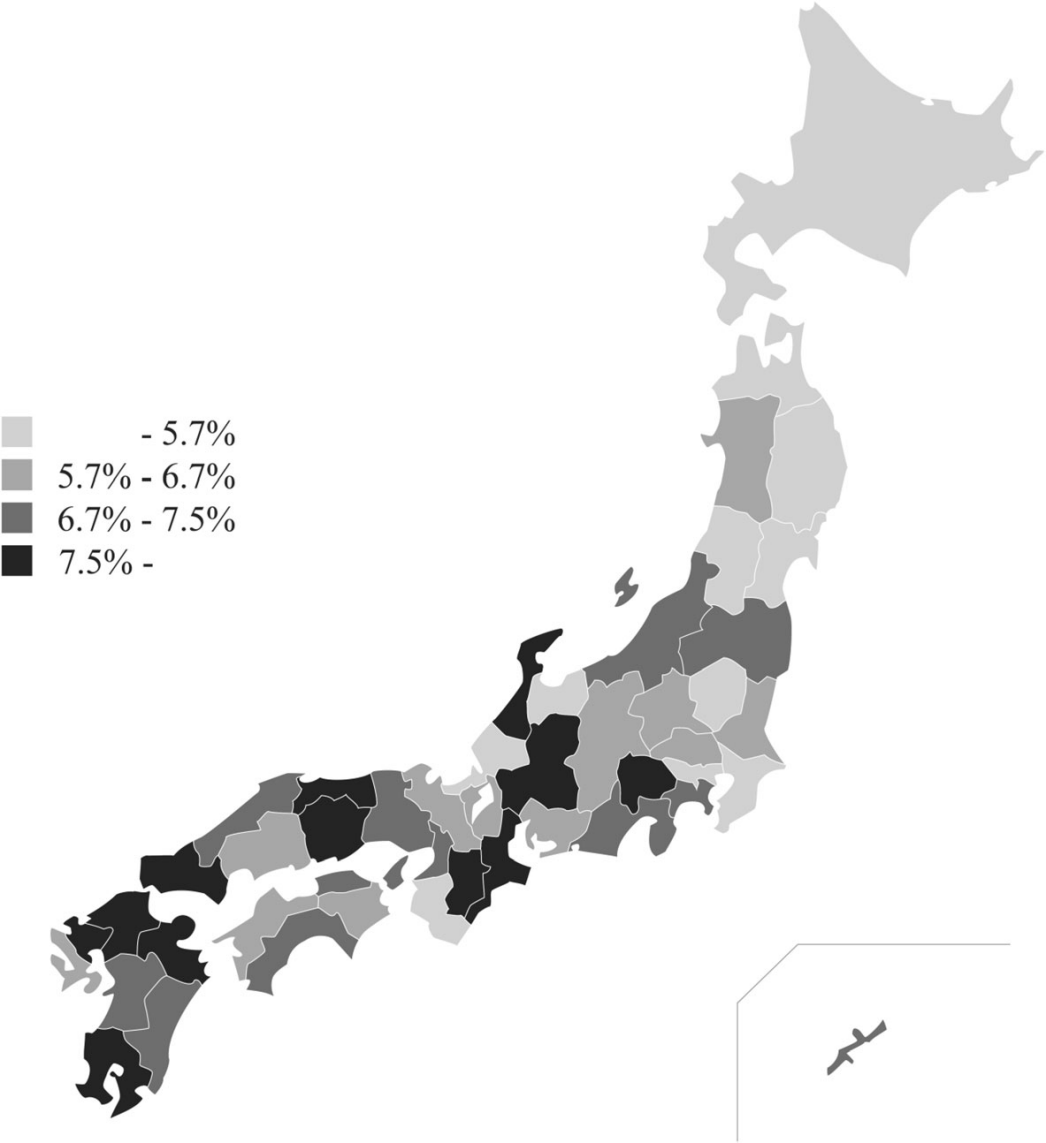
Prevalence of MRSA in prefectures for annual Open Report 2015

Table 2 shows the trend in AMR prevalence between 2008 and 2015, according to the JANIS Annual Open Reports. One of the most prevalent AMR bacteria in Japan is MRSA, which was detected in about 10% of clinical samples submitted for microbiological tests in 2008. However, its prevalence decreased constantly, dropping below 7% in 2014. There was also a geographical difference in the prevalence of MRSA, as shown in Fig. 5, indicating higher prevalence in the western part of Japan.

**Table 2:** The trend of prevalence of AMR during 2008 to 2015

Type of AMR	2008	2009	2010	2011	2012	2013	2014	2015
Methicillin-resistant *Staphylococcus aureus*	10.46%	10.01%	9.43%	8.77%	8.06%	7.48%	6.91%	6.64%
Vancomycin-resistant Enterococci	0.03%	0.05%	0.05%	0.03%	0.02%	0.02%	0.02%	0.02%
Penicillin-resistant *Streptococcus pneumoniae*	1.31%	1.29%	1.38%	1.15%	0.89%	0.79%	0.69%	0.64%
Multidrug-resistant *Pseudomonas aeruginosa*	0.23%	0.18%	0.18%	0.18%	0.14%	0.12%	0.09%	0.07%
Multidrug-resistant *Acinetobacter* spp.	0.00%	0.00%	0.01%	0.01%	0.01%	0.01%	0.01%	0.01%
Carbapenem-resistant Enterobacteriaceae	ND	ND	ND	ND	ND	ND	0.49%	0.36%
Carbapenem-resistant *Pseudomonas aeruginosa*	1.45%	1.30%	1.26%	1.26%	1.09%	0.98%	0.88%	0.84%
3rd generation cephalosporin-resistant *Klebsiella pneumoniae*	0.17%	0.18%	0.19%	0.24%	0.24%	0.23%	0.33%	0.32%
3rd generation cephalosporin-resistant *Escherichia coli*	0.62%	0.70%	0.86%	1.14%	1.30%	1.40%	1.82%	1.99%
Fluoroquinolone-resistant *Escherichia coli*	1.79%	1.88%	2.15%	2.52%	2.87%	3.12%	3.35%	3.70%
Patients for whom samples were submitted	930,861	1,056,555	1,069,216	1,309,993	1,453,969	1,584,041	1,747,538	2,551,541

### Feedback reports of JANIS

**Fig. 6:**
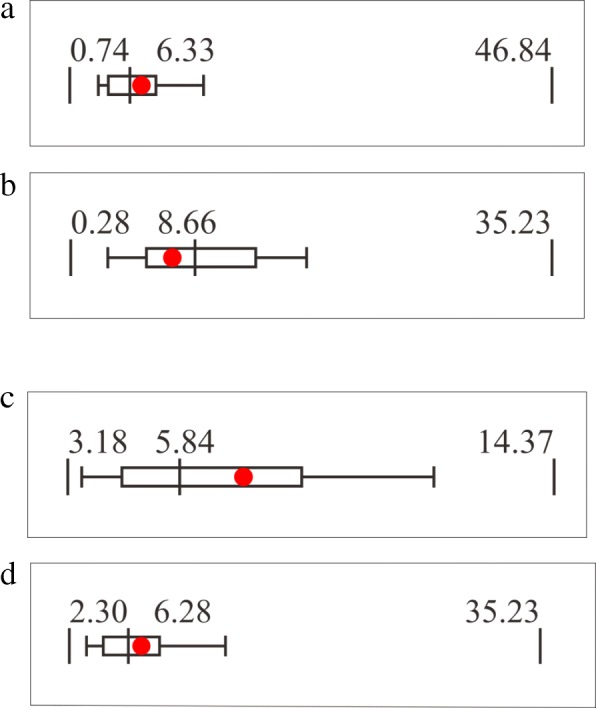
**a**) JANIS box plot chart from Feedback Reports of a large hospital with ≥200 beds and a MRSA prevalence of 7.39%. **b**) JANIS box plot chart from Feedback Reports of a small hospital with < 200 beds and a MRSA prevalence of 7.39%. **c**) and **d**) JANIS box plot charts from Feedback Reports of two hospitals that have the same MRSA prevalence (7.39%) but are located in different prefectures

Figure 6a and b show JANIS box plot charts of MRSA from 2015 Feedback Reports of two hospitals that had different bed counts but identical prevalence of MRSA (7.39%). Figure 6a is from a hospital with ≥200 beds (large hospital), and Fig. 6b is from a hospital with < 200 beds (small hospital). When stratified by hospital bed counts, the prevalence of MRSA in small hospitals is higher than that in large hospitals (9.37% vs 6.50%, respectively). Therefore, the same prevalence may be interpreted differently when hospital type is taken into account. A MRSA prevalence of 7.39% is relatively high for a large hospital, and may require interventions for infection control, but the same prevalence in a small hospital can be interpreted as well controlled. The same phenomenon was observed when the data were stratified by prefecture. Figure 6c and d are Feedback Reports based on data stratified by 47 prefectures, with hospitals in which the MRSA prevalence was also 7.39% but that were located in different prefectures.

## Discussion

From the beginning, JANIS was designed to collect comprehensive bacterial data from hospital diagnostic microbiology laboratories even though it was initially limited to blood and CSF samples. This seemed to be a promising approach because both automated microbiology test systems and laboratory information systems became widely used in hospitals during early 2000’s. Hospitals already have the electronic data from their routine bacteriological tests, which are supposed to be submitted monthly to JANIS.

Data collection focusing on blood and CSF samples minimizes heterogeneity and improves comparability between reporting laboratories but information would be limited to invasive infection [15]. European Antimicrobial Resistance Surveillance Network (EARS-Net) and Central Asian and Eastern European Surveillance of Antimicrobial Resistance focus on blood and CSF samples [8, 16]. In UK, antimicrobial resistance surveillance on routinely generated laboratory results has started in 1989, including the mandatory reporting of MRSA bacteremia to the Public Health England and its predecessors [7, 17, 18]. However, WHO Global Antimicrobial Resistance Surveillance System (GLASS) recommends comprehensive data collection at the national level to include other species and specimen types beyond their priority list [2].

During the first several years after the JANIS CL division was launched, half of the enrolled hospitals did not submit surveillance data. This failure may have been due to the insufficient announcement of surveillance methods, delayed or poor-quality Feedback Reports which were printed out and sent by mail 6 to 12 months after data submission, and limited internet access during that time period.

The revision of JANIS in 2007 was intended to settle these issues, and it was possible partly because most of the hospitals had improved their internet access by the middle of the 2000s. As a result of those systemic revisions and annual recruitment of participants, the number of member hospitals increased year by year, even though JANIS is a voluntary surveillance system. The inclusion of participation in the JANIS CL division as a prerequisite for receiving additional reimbursement for infection control demonstrates that JANIS has been established as a sustainable national surveillance system.

Although the number of participating hospitals has been increased, the majority of participants are large hospitals, and only about 4% of small hospitals with less than 200 beds were covered by the JANIS CL division in 2015. Thus, JANIS CL division data may over-represent AMR epidemiology of large hospitals in Japan. This may be because small hospitals usually do not have their own microbiology laboratories and their subscribing commercial laboratories may not provide surveillance data. In addition, in the absence of their own microbiology laboratories, these hospitals are unlikely to employ professionals who can interpret and utilize feedback from the JANIS CL division. How to collect and analyze surveillance data from those small hospitals is an important issue for JANIS to address in the future, and these questions are also critical for AMR surveillance systems in general. The EARS-Net also pointed out the over-representation of large hospitals, and also noted that tertiary care hospitals are much more likely to participate in this surveillance than smaller hospitals [19].

In addition, even if the coverage of small hospitals were increased, interpretation of surveillance data requires careful consideration of the denominator due to variability in the frequency of culture sampling. The Society for Healthcare Epidemiology and the Healthcare Infection Control Practices Advisory Committee suggests calculating prevalence of AMR per 100 patients or 1000 patient-days admitted to the hospital [20]. However, because AMR can only be detected by specimen submission, JANIS calculates the prevalence of AMR by defining the denominator as the number of patients for whom specimens were submitted. Moreover, one of the metrics to measure the occurrence of AMR proposed by WHO GLASS is to have the total number of sampled patients per specimen type as the denominator. To our knowledge, JANIS is the only national surveillance that truly collects and analyzes comprehensive data, including active surveillance testing and culture-negative results for all specimens [21]. Data for all specimens can provide information about how samples were submitted from each hospital. These data revealed that the number of samples per 100 beds for small hospitals is half of that of large hospitals, and more sterile samples such as blood cultures are obtained in large hospitals, whereas more easy-to-obtain samples (such as respiratory and urine samples) are collected in small hospitals. Inversely, the proportion of culture-positive specimens of small hospitals is higher than that of large hospitals, indicating minimal bacterial investigation in small hospitals, which may overestimate the prevalence of AMR. The higher prevalence of MRSA in the western part of Japan may partially be explained by that there are more small hospitals per 100,000 capita in that region, and the sampling frequency is low in that type of hospital.

Quality assurance of diagnostic microbiology laboratories and data validation is fundamental to AMR surveillance, especially for a system that collects data but not bacterial isolates to be tested in a central reference laboratory. One reason that JANIS CL division initially targeted only large hospitals was to ensure data reliability, as those hospitals are expected to employ clinical microbiology professionals. The diffusion of automated microbiology test systems among hospital laboratories in Japan guarantees the basic quality of microbiological tests because the manufacturers of those systems provide quality assurance services to the custom hospitals. There are a few domestic external quality assessment (EQA) programs in which most of the JANIS member hospitals with in-house laboratories may be enrolled. Large commercial laboratories are also enrolled in internationally approved EQA, but those programs are difficult to afford for hospitals and small commercial laboratories. Accordingly, a national scheme for laboratory quality assurance is required. Data validation for Unusual AMR by the JANIS CL division could play a certain role in quality assurance because it would inform hospitals what type of microbiology test results requires confirmation.

The accumulation of nationwide surveillance data over a decade enables us to describe the long-term trends in AMR prevalence. There are encouraging findings that AMR of major clinically important bacterial species in Japan is decreasing or maintained at a low level. For instance, the prevalence of MRSA and multidrug-resistant *Pseudomonas aeruginosa* has been decreasing since 2008, to around half (or less) of the previous levels. It is also notable that multidrug-resistant *Acinetobacter* spp. (MDRA), which are prevalent in many Asian countries, are still well controlled in Japan [22]. On the other hand, antimicrobial resistance among *Escherichia coli* keeps increasing as many other countries.

Even though the principal purpose of JANIS is to create national AMR profiles, it was initiated to support infection prevention and control in medical facilities. Feedback Reports to each participating hospital are created to raise awareness of infection control practices and to motivate data submission through the box plot chart, which intuitively benchmarks the prevalence of AMR of each facility against the entire distribution. To enable this benchmarking, JANIS collects MIC values rather than interpreted RIS because there are different standards and frequent updates for the categorization of RIS. In addition, JANIS applies a unified de-duplication program to exclude repeated isolates from the same patients.

Stratification by hospital bed counts and prefectures revealed different distributions of AMR prevalence among hospital types and geographical regions. This indicates that the goals of AMR containment should be set in consideration of local factors, and that regional cooperation is essential. In fact, several regional networks share JANIS files to create their local antibiograms.

There are several limitations of the JANIS CL division, suggesting opportunities for improvement. WHO GLASS requires data on AMR combined with patient and microbiological information. All of them are included as data elements in JANIS data format but currently clinical data are rarely reported because of the different data sources. If integration of patient and microbiological information in each facility became more feasible, it may become possible to fulfil GLASS requirement.

Inclusion of data from small hospitals is challenging, but collection of such data has been gradually increasing; in 2017, 8.5% of 5800 small hospitals are participating in JANIS, almost double the number from 2015. Population-based AMR surveillance, which captures AMR data from an entire predefined population or a representative sample, is considered to be the gold standard for surveillance [4]. However, laboratory-based surveillance covering a small number of sentinel sites (fewer than 20% of national hospitals) is considered to be sufficient to provide relatively robust estimates of national AMR prevalence, assuming that the sampling is constant and the sample is representative of the target population [15]. Therefore, increasing sampling frequency, especially prior to treatment with antibiotics, may increase the representativeness of the JANIS Open Report for small hospitals more effectively than recruiting additional hospitals. Moreover, data validation is a challenge for small hospitals without microbiology staff. Accordingly, support of regional hospital networks is required for purposes of education.

## Conclusions

JANIS achieved sustainability as a national AMR surveillance by initially focusing on large hospitals with diagnostic microbiology laboratories in order to establish the management scheme of the surveillance. Comprehensive data for all specimens promotes understanding of the sampling frequency and prevalence of AMR. Because JANIS is a well-established system for providing rich information to guide action both locally and nationally, it may also be utilized for sharing AMR data globally.

## Additional files

Additional file 1: Data format of JANIS Clinical Laboratory division. -All data fields are fixed-length. If the input data string is shorter than the specified length, the remainder needs to be filled with single-byte spaces (i.e., 0 × 20 or “”). Data requirements: “M”, mandatory; “S”, suggested. (M) and (S) indicate that the field may be left blank if the data are not relevant. (DOCX 33 kb)

Additional file 2: Interpretive Criteria for Specific AMR Bacteria. (DOCX 31 kb)

Additional file 3: Definition of Unusual AMR bacteria. Category A: AMR bacteria never reported in Japan. Category B: AMR bacteria rarely reported in Japan. RIS interpretation is based on the CLSI 2012 (M100-S22) criteria. † Criteria are based on the Infectious Diseases Control Law. (DOCX 28 kb)

Additional file 4: Data validation criteria for annual Open Report 2015. (DOCX 26 kb)

## Abbreviations

AMR: Antimicrobial resistance
AST: Antimicrobial susceptibility testing
CL: Clinical Laboratory
CLSI: Clinical and Laboratory Standards Institute
CSF: Cerebrospinal fluids
EARS-Net: European Antimicrobial Resistance Surveillance network
JANIS: Japan Nosocomial Infections Surveillance
MHLW: Ministry of Health, Labour and Welfare
MICs: Minimal inhibitory concentrations
MRSA: methicillin-resistant *Staphylococcus aureus*
RIS: Resistant, intermediate, or susceptible
