# Supplementary material for: Japan nosocomial infections surveillance (JANIS): a model of sustainable national antimicrobial resistance surveillance based on hospital diagnostic microbiology laboratories
Source: BMC Health Serv Res. 2018 Oct 20;18:799. doi: 10.1186/s12913-018-3604-x (PMC6195991; doi:10.1186/s12913-018-3604-x)
Supplement: Supplementary file 1 — Data format of JANIS Clinical Laboratory division. -All data fields are fixed-length. If the input data string is shorter than the specified length, the remainder needs to be filled with single-byte spaces (i.e., 0 × 20 or “”). Data requirements: “M”, mandatory; “S”, suggested. (M) and (S) indicate that the field may be left blank if the data are not relevant. (DOCX 33 kb) [file 12913_2018_3604_MOESM1_ESM.docx]

#

|  | Data requirement | No | Item | Attribute | Length | Starting Location | Specification |
| --- | --- | --- | --- | --- | --- | --- | --- |
| Basic Patient Data |  | 1 | Surveillance Category | numeric | 1 | 1 | 1: Laboratory Division, 2: National Hospital, 3: ICU |
|  | M | 2 | Facility Code | numeric | 5 | 2 | Province Code 2 digit、Prefecture serial number 3 digit |
|  | M | 3 | Patient ID | alphanumeric/symbol | 15 | 7 | Encrypted unique ID using 15 single-byte characters |
|  |  | 4 | Date of Admission | numeric | 8 | 22 | YYYY; year MM ; month DD; day |
|  | S | 5 | Sex | alphabet | 1 | 30 | M: Male, F: Female |
|  | S | 6 | Date of Birth | numeric | 8 | 31 | YYYY; year MM ; month DD; day |
|  | M | 7 | Hospital Status | numeric | 1 | 39 | 1:Outpatient, 2:Inpatient |
|  | S | 8 | Department | numeric | 3 | 40 | Refer to "Department Code" |
|  | S | 9 | Ward | alphanumeric | 15 | 43 | Free text format（alphanumeric only） |
|  |  | 10 | Blank | space | 409 | 58 |  |
| Specimen Data | M | 11 | Specimen Source | numeric | 3 | 467 | Refer to "Specimen Source Code" |
|  | M | 12 | Specimen Reception Date | numeric | 8 | 470 | YYYY; year MM ; month DD; day |
|  | S | 13 | Specimen Collection Date | numeric | 8 | 478 | YYYY; year MM ; month DD; day |
|  |  | 14 | Blank | space | 13 | 486 |  |
|  | S | 15 | Bacterial Culture Result | 1 or space | 1 | 499 | 1: negative, space: positive |
| Bacterial Test Data | (M) | 16 | Isolated Bacterium "A" | numeric | 4 | 500 | Refer to "Isolated Bacterial code" |
|  | (S) | 17 | Quantification of Bacterial Amount | numeric | 1 | 504 | 1:semi-quantitative analysis 2:quantitative analysis 9:others |
|  | (S) | 18 | Amount of Bacterium "A" | numeric | 1 | 505 | 1:≦10^2/ml, 2:10^3/ml, 3:10^4/ml, 4:10^5/ml, 5:10^6/ml, 6:≧10^7/ml, 7:10^3-10^4/ml, 8:10^5-10^6/ml |
|  |  | 19 | Blank | space | 1 | 506 |  |
|  | (M) | 20 | Isolated Bacterium "B" | numeric | 7 | 507 |  |
|  | (M) | 24 | Isolated Bacterium "C" | numeric | 7 | 514 |  |
|  | (M) | 28 | Isolated Bacterium "D" | numeric | 7 | 521 |  |
|  | (M) | 32 | Isolated Bacterium "E" | numeric | 7 | 528 |  |
|  | (M) | 36 | A-1 Antimicrobial Agent | numeric | 4 | 535 | Refer to "Antimicrobial Code" |
|  | (M) | 37 | A-1 Susceptibility Test Method | numeric | 2 | 539 | Refer to "Susceptibility Test Method Code" |
|  | (M) | 38 | A-1 Sign | numeric | 1 | 541 | 1: <, 2: >, 3: ≦, 4: ≧, space: = |
|  | (M) | 39 | A-1 MIC value | numeric/period | 5 | 542 | XXXXX （right-adjusted integer） or X.XXX（fixed decimal point format） |
|  | (S) | 40 | A-1 Zone Diameter | numeric | 2 | 547 | XX (millimeters in integer) |
|  | (M) | 41 | A-1 Interpretation (RIS) | alphabet | 1 | 549 | R: resistant, I: intermediate, S: susceptible |
|  | (M) | 42 | A-1 Interpretation(+) | numeric | 1 | 550 | 1:-, 2:+, 3:++, 4:+++ |
|  | (M) | 43 | A-2 to A-30 |  | 464 | 551 |  |
|  | (M) | 246 | B |  | 480 | 1015 |  |
|  | (M) | 456 | C |  | 480 | 1495 |  |
|  | (M) | 666 | D |  | 480 | 1975 |  |
|  | (M) | 876 | E |  | 480 | 2455 |  |
| Other Data |  | 1086 | Blank | space | 24 | 2935 |  |
|  | M | 1087 | Specimen ID | alphanumeric/hyphen | 15 | 2959 | Assign a unique identifier for each specimen |
|  |  | 1088 | Blank | space | 23 | 2974 |  |
|  | M | 1089 | Version ID | alphanumeric/period | 4 | 2997 | Refer to "Handbook for the JANIS Clinical Laboratory Division (JCLD) Data Submission" |
|  |  | 1090 | Blank | space | 50 | 3001 |  |
