# Supplementary material for: Japan nosocomial infections surveillance (JANIS): a model of sustainable national antimicrobial resistance surveillance based on hospital diagnostic microbiology laboratories
Source: BMC Health Serv Res. 2018 Oct 20;18:799. doi: 10.1186/s12913-018-3604-x (PMC6195991; doi:10.1186/s12913-018-3604-x)
Supplement: Supplementary file 2 — Interpretive Criteria for Specific AMR Bacteria. (DOCX 31 kb) [file 12913_2018_3604_MOESM2_ESM.docx]

| Isolated Bacterium | Comments* | MIC Value | Isolated Bacterial Code |
| --- | --- | --- | --- |
|  |  |  |  |
| Methicillin-Resistant *Staphylococcus aureus* (MRSA) | *S. aureus* resistant to oxacillin by the broth microdilution method or methicillin-resistant *S. aureus* detected on selective media | oxacillin ≥ 4 μg/ml | 1301, 1303 |
|  |  |  |  |
| Vancomycin-Resistant *Staphylococcus aureus* (VRSA) | *S. aureus* resistant to vancomycin | vancomycin ≥ 16 μg/ml | 1301,1303–1306 |
|  |  |  |  |
|  |  |  |  |
| Vancomycin-Resistant Enterococci (VRE) | *Enterococcus* spp*.* resistant† to vancomycin by the broth microdilution method or vancomycin-resistant enterococci detected on selective media | vancomycin ≥ 16 μg/ml† | 1201,1202,1205, |
|  |  |  | 1206,1209,1210, |
|  | Note: Excluding species that were not identified within *Enterococcus* sp. |  | 1213–1217 |
| Penicillin-Resistant *Streptococcus pneumoniae* (PRSP) | *S. pneumoniae* resistant† to benzylpenicillin | benzylpenicillin ≥ 0.125 μg/ml† | 1131 |
|  |  |  |  |
| Multidrug-Resistant *Pseudomonas aeruginosa* (MDRP) | *P. aeruginosa* satisfying all of the following criteria: |  | 4001 |
|  | 1. Resistant† to carbapenems (imipenem and/or meropenem) | 1. imipenem ≥ 16 μg/ml†, meropenem ≥ 16 μg/ml† |  |
|  | 2. Resistant† to aminoglycosides (amikacin) | 2. amikacin ≥ 32 μg/ml† |  |
|  | 3. Resistant to fluoroquinolones (any of norfloxacin, ofloxacin, levofloxacin, ciprofloxacin, lomefloxacin, and gatifloxacin). | 3. norfloxacin ≥ 16 μg/ml, |  |
|  |  | ofloxacin ≥ 8 μg/ml, |  |
|  |  | levofloxacin ≥ 8 μg/ml, |  |
|  |  | lomefloxacin≥ 8 μg/ml, |  |
|  |  | gatifloxacin ≥ 8 μg/ml, |  |
|  |  | ciprofloxacin ≥ 4 μg/ml |  |
| Multidrug-Resistant *Acinetobacter* spp. (MDRA) | *Acinetobacter* spp. satisfying all of the following criteria: |  | 4400–4403 |
|  | 1. Resistant to carbapenems (imipenem and/or meropenem) | 1. imipenem ≥ 16 μg/ml†, meropenem ≥ 16 μg/ml† |  |
|  | 2. Resistant† to aminoglycosides (amikacin) | 2. amikacin ≥ 32 μg/ml† |  |
|  | 3. Resistant to fluoroquinolones (any of levofloxacin, ciprofloxacin, and gatifloxacin). | 3. levofloxacin ≥ 8 μg/ml, |  |
|  |  | ciprofloxacin ≥ 4 μg/ml, |  |
|  |  | gatifloxacin ≥ 8 μg/ml |  |
| Carbapenem-Resistant Enterobacteriaceae (CRE) | Enterobacteriaceae satisfying one of the following criteria. |  | 2000-2394, 3150-3151 |
|  | 1. Resistant† to meropenem | meropenem ≥ 16 μg/ml† |  |
|  | 2. Resistant† to imipenem and also resistant to cefmetazole | imipenem ≥ 16 μg/ml†, and also |  |
|  |  | cefmetazole ≥ 64 μg/ml |  |
| Carbapenem-Resistant *Pseudomonas aeruginosa* | *P. aeruginosa* resistant to imipenem and/or meropenem | imipenem ≥ 16 μg/ml†, | 4001 |
|  |  | meropenem ≥ 16 μg/ml† |  |
| 3rd Generation Cephalosporin-Resistant *Klebsiella pneumoniae* | *K. pneumoniae* resistant to cefotaxime and/or ceftazidime | cefotaxime ≥ 16 μg/ml, | 2351 |
|  |  | ceftazidime ≥ 32 μg/ml |  |
| 3rd Generation Cephalosporin-Resistant *Escherichia coli* | *E. coli* resistant to cefotaxime and/or ceftazidime | cefotaxime ≥ 16 μg/ml, | 2001–2007 |
|  |  | ceftazidime ≥ 32 μg/ml |  |
| Fluoroquinolone-Resistant *Escherichia coli* | *E. coli* resistant to fluoroquinolones (any of norfloxacin, ofloxacin, levofloxacin, lomefloxacin, gatifloxacin, and ciprofloxacin) | norfloxacin ≥ 16 μg/ml, | 2001–2007 |
|  |  | ofloxacin ≥ 8 μg/ml, |  |
|  |  | levofloxacin ≥ 8 μg/ml, |  |
|  |  | lomefloxacin ≥ 8 μg/ml, |  |
|  |  | gatifloxacin ≥ 8 μg/ml, |  |
|  |  | ciprofloxacin ≥ 4 μg/ml |  |
