# Supplementary material for: Japan nosocomial infections surveillance (JANIS): a model of sustainable national antimicrobial resistance surveillance based on hospital diagnostic microbiology laboratories
Source: BMC Health Serv Res. 2018 Oct 20;18:799. doi: 10.1186/s12913-018-3604-x (PMC6195991; doi:10.1186/s12913-018-3604-x)
Supplement: Supplementary file 3 — Definition of Unusual AMR bacteria. Category A: AMR bacteria never reported in Japan. Category B: AMR bacteria rarely reported in Japan. RIS interpretation is based on the CLSI 2012 (M100-S22) criteria. † Criteria are based on the Infectious Diseases Control Law. (DOCX 28 kb) [file 12913_2018_3604_MOESM3_ESM.docx]

Category A:

| Isolated bacterial code | Bacterial name | Antimicrobial code | Drug name | RIS interpretation | MIC value |
| --- | --- | --- | --- | --- | --- |
| 1111 | *Streptococcus pyogenes* | 1201 | Benzylpenicillin | NS | > 0.12μg/ml |
|  |  | 1216 | Ampicillin | NS | > 0.25μg/ml |
|  |  | 2301 | Vancomycin | NS | > 1μg/ml |
|  |  | 2616 | Linezolid | NS | > 2μg/ml |
| 1114 | *Streptococcus agalactiae* | 2301 | Vancomycin | NS | > 1μg/ml |
|  |  | 2616 | Linezolid | NS | > 2μg/ml |
| 1131 | *Streptococcus pneumoniae* | 2301 | Vancomycin | NS | > 1μg/ml |
|  |  | 2616 | Linezolid | NS | > 2μg/ml |
| 1301, 1303-1306 | *Staphylococcus aureus* | 2301 | Vancomycin | R | ≧16μg/ml |

Category B:

| Isolated bacterial code | Bacterial name | Antimicrobial code | Drug name | RIS interpretation | MIC value |
| --- | --- | --- | --- | --- | --- |
| 1201-1202, 1205-1206 | *Enterococcus faecalis Enterococcus faecium* | 2301 | Vancomycin | R† | ≧16μg/ml |
|  |  | 2306 | Teicoplanin | I, R | ≧16μg/ml |
|  |  | 2616 | Linezolid | R | ≧8μg/ml |
| 1301, 1303-1306 | *Staphylococcus aureus* | 2306 | Teicoplanin | I, R | ≧16μg/ml |
|  |  | 2616 | Linezolid | R | ≧8μg/ml |
| 1311-1325 | Staphylococcus, coagulase negative (CNS) | 2301 | Vancomycin | I, R | ≧16μg/ml |
| 4400-4403 | *Acinetobacter* spp. fulfill all the conditions on the right | 1401 | Imipenem | R | ≧16μg/ml |
|  |  | 1411 | Meropenem |  |  |
|  |  | 1816 | Amikacin | R† | ≧32μg/ml |
|  |  | 2521 | Ciprofloxacin | R | ≧4μg/ml |
|  |  | 2516 | Levofloxacin |  | ≧8μg/ml |
|  |  | 2561 | Gatifloxacin |  | ≧8μg/ml |
