# Supplementary material for: Japan nosocomial infections surveillance (JANIS): a model of sustainable national antimicrobial resistance surveillance based on hospital diagnostic microbiology laboratories
Source: BMC Health Serv Res. 2018 Oct 20;18:799. doi: 10.1186/s12913-018-3604-x (PMC6195991; doi:10.1186/s12913-018-3604-x)
Supplement: Supplementary file 4 — Data validation criteria for annual Open Report 2015. (DOCX 26 kb) [file 12913_2018_3604_MOESM4_ESM.docx]

| ✔ | No inpatient specimen data reported throughout the year. | |
| --- | --- | --- |
| ✔ | No *Staphylococcus aureus*, methicillin-resistant *Staphylococcus aureus* (MRSA) and *Escherichia coli* reported throughout the year. | |
| ✔ | No blood, urine, and respiratory specimen reported throughout the year. | |
| ✔ | Blood culture positivity rate above or equal to 50% to check outliers, for facilities reporting more than 10 blood cultures per year. | |
| ✔ | Cerebrospinal fluid culture (CSF) positivity rate above or equal to 50%, for facilities reporting more than 5 CSF cultures per year. | |
| ✔ | More than one report of Category A: AMR bacteria never reported in Japan | |
| ✔ | No data tested by broth microdilution method |  |
| ✔ | Carbapenem-resistant Enterobacteriaciae isolation rate† more than or equal to 5% to check outliers. | |
|  | † Isolation rate = (Number of patients with CRE for each facility) / (Number of specimen-submitting patients for each facility)×100 | |
